# Air sensitivity of GaSe 2D material and its potential implications on device reliability

**DOI:** 10.1186/s11671-026-04568-9

**Published:** 2026-04-17

**Authors:** Hazel Neill, Lida Ansari, Vilas Patil, Stephen O’Sullivan, Brendan Roycroft, Martina Piletti, Daniela Iacopino, Paul K. Hurley, Farzan Gity

**Affiliations:** 1 Tyndall National Institute, University College Cork, Lee Maltings, Dyke Parade, Cork, T12 R5CP Ireland; 2 School of Chemistry, University College Cork, Cork, Ireland

## Abstract

This study demonstrates the oxidation-driven degradation of exfoliated GaSe highlighting the changes to not only the material’s morphology but its chemical composition as well. We have employed a number of simulation and experimental techniques, including DFT calculations, SEM, EDX and Raman spectroscopy, to understand the atomic structure and vacancy effects between the β and ε polytypes, and to understand the degradation occurring. The combined results of the DFT and experimental results show that the crystal used was the β polytype with a bandgap value of ~ 1.92 eV. Further experimental analysis demonstrates that the oxidation occurs on the surface of the material producing Ga_2_O_3_ which is possibly due to defect sites or dangling bonds. This oxidation encourages the formation of hemispherical blisters on the material surface. EDX analysis indicates that these blisters are Se-rich while Raman spectroscopy confirms the presence of amorphous and crystalline Se in these regions while also showing the disappearance of the dominant vibrational modes of GaSe. This work emphasises the importance of the environment’s role and developing encapsulation strategies in relation to interface engineering and material stability for fabricating air-stable nanoelectronic devices.

**Supplementary Information:**

The online version contains supplementary material available at 10.1186/s11671-026-04568-9.

## Introduction

It is well known that two-dimensional (2D) materials are air sensitive, this is the case for transition metal dichalcogenides (TMDs) and layered monochalcogenides, especially GaSe as presented in this work. 2D materials have attracted significant attention by the scientific community and semiconductor manufacturing industry due to their potential exotic transport physics and technological applications in various fields including a significant device downscaling for high intensity integration. Recently, a variety of 2D materials has been explored, pioneered by graphene-based field‐effect transistors (FET), for their potential use in logic and memory applications [1–3]. The main advantage of graphene‐based FETs is the associated carrier high mobility of up to 10^6^ cm^2^/Vs; however, the lack of bandgap and therefore low on/off ratio in graphene‐based electronics has created a motivation to explore other 2D materials beyond graphene, including MoS_2_, PtSe_2_ and WS_2_ which have been experimentally and theoretically investigated, demonstrating great promise for low-power, high performance electronics applications [4–6]. Although most research has focused on 2D transition metal dichalcogenides (TMDs), recently 2D layered metal monochalcogenides, such as GaS, InSe and especially GaSe have attracted increasing interest due to their significantly different electronic and optoelectronic properties compared to TMDs [7–14].

Gallium selenide (GaSe) is a layered 2D binary chalcogenide material belonging to the III-VI group. GaSe has similar structural properties to other 2D materials, such as TMDs, as each layer is formed through the covalent bonds between the Ga and Se atoms and these layers are held together with relatively weak van der Waals interactions. There has been significant research into GaSe due to its direct band gap ~ 2.1 eV leading to its applications in optoelectronics, photodetectors and solar cells [15–17]. As a lamellar material, GaSe can be grown in large-scale [18], while also being exfoliated, further promoting its applications to van der Waal heterostructures. Bulk GaSe crystal structure which has a hole mobility ≈ 215 cm^2^/V.s [8, 19, 20] has hexagonal symmetry [8, 21] and comprises of vertically stacked Ga-Se-Se-Ga layers. GaSe has four polytypes that can form the crystallographic structure, ε-(2R), β-(2H), γ-(3R), and δ-(4 H) [22–24]. The two main GaSe polytypes which differ in the stacking sequence of the base layer units, i.e., β-GaSe and ε-GaSe, are examined in more detail in this study.

GaSe shows many interesting electrical and optical properties and has been widely used in the fields of optoelectronics/phototransistors, Schottky-junction based FETs, (chemiresistor) gas sensors, spintronics, nonlinear optics and broadband terahertz wave detection [11, 25–35]. Recently, the photoresponse of exfoliated [8, 11, 36], vapor phase deposited [12, 37], and metal-organic chemical vapour deposited [7, 38] GaSe has been reported showing their potential properties in optoelectronic devices. Furthermore, theoretical studies have predicted that the bandgap of GaSe may be widely tuned by varying the number of layers in the crystal due to quantum confinement or by the inducing mechanical strain [8, 11, 37, 39–43].

Similar to other chalcogenides, such as HfSe_2_, MoTe_2_ and GeS, GaSe experiences degradation over time when exposed to ambient air [43–46]. The degradation is caused by oxidation of the top layers of the material, leading to the formation of Ga_2_O_3_ and Ga_2_Se_3_ as well as Se-rich particles [44–46]. These particles which appear as hemispherical protrusions on the surface occur at a faster oxidation rate on exfoliated samples due to the dangling Se bonds created when the van der Waal interactions between layers were broken during exfoliation [45]. The oxidation of GaSe reduces the effective film layer thickness of the material and impacts its properties [47], especially with thinner flakes ~8 L in which oxidation has been reported within a few hours of exfoliation [48].

## Results and discussion

### Electronic structure of GaSe

GaSe has several polytypes - structural variants that arise from different stacking sequences of its layered units. While these polytypes share the same chemical composition, they differ in crystal symmetry, stacking order, and the number of layers per unit cell, which in turn influences their electronic, optical, and mechanical properties [49–51]. Among them, β-GaSe (2 H) is the most common and thermodynamically stable polytype at room temperature [52], characterized by its high symmetry and hexagonal layered structure. In contrast, ε-GaSe (3R) is a metastable phase that can form under strain or specific growth conditions.

**Fig. 1:**
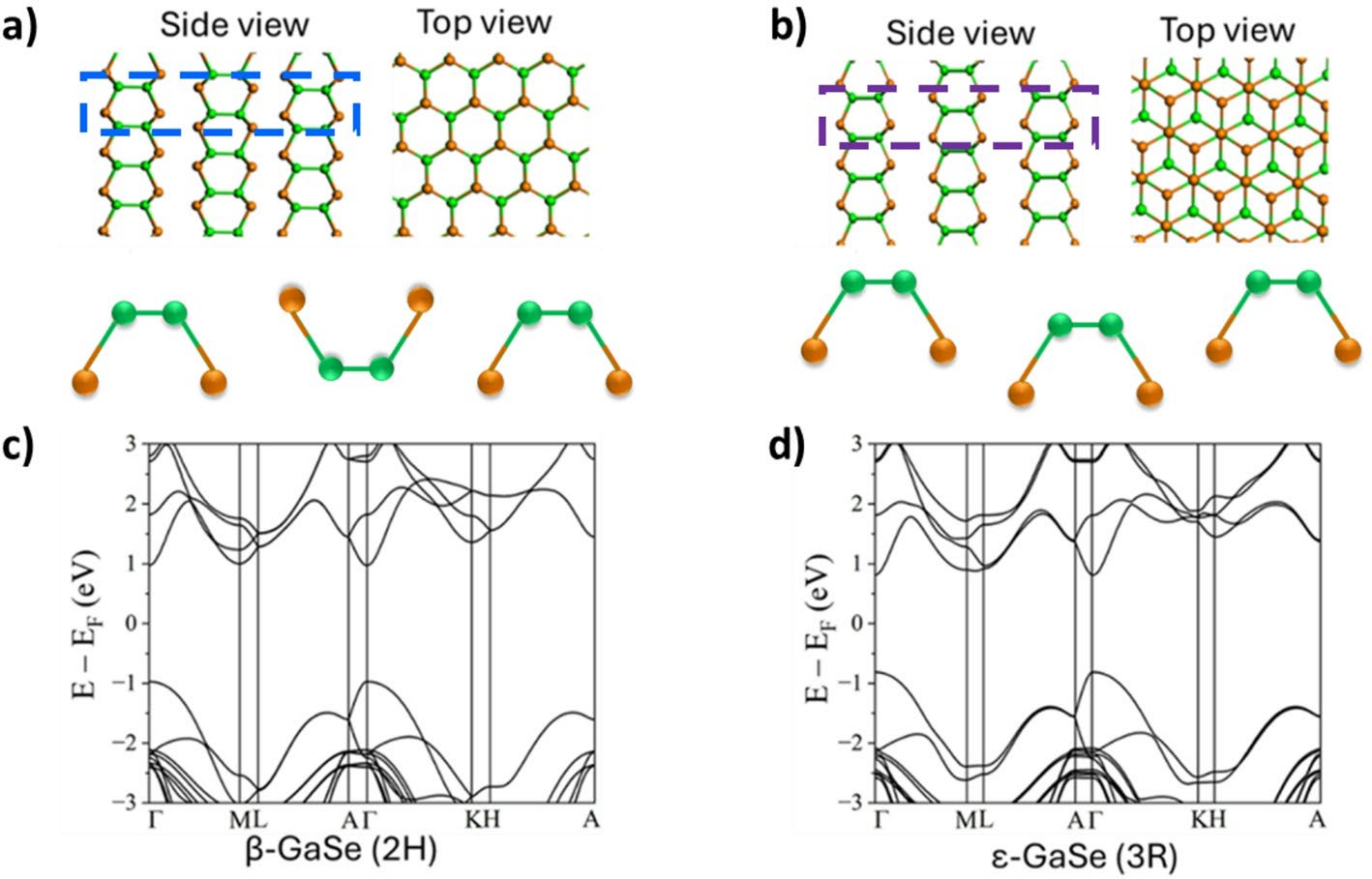
Atomic structures of **a** β-GaSe and **b** ε-GaSe polytypes. The blue and purple dashed boxes highlight the different orientation of the bonds responsible for the β-GaSe and ε-GaSe polytypes, respectively. In the atomic models, orange spheres represent selenium (Se) atoms and green spheres represent gallium (Ga) atoms. The bottom row shows the corresponding electronic band structures for **c** β-GaSe and **d** ε-GaSe, post GGA-1/2 band gap calibration.

Figure 1 illustrates the atomic structures and corresponding electronic band structures of these two GaSe polytypes. In the atomic models (panels a and b), orange atoms represent selenium (Se), and green atoms represent gallium (Ga). The different stacking sequences result in distinct interlayer interactions and slight variations in bond angles, which contribute to the differences in their electronic band dispersion. Our GGA-1/2-calibrated (see Methods Section) calculations yield band gap energies of 2 eV and 1.7 eV for β-GaSe and ε-GaSe, respectively, which are in good agreement with our experimental results (See Fig. S1 in the Supplementary Information (SI)) and with previously reported values in the literature [49]. Figure 1c and d further highlight the differences in the electronic band structures of the two polytypes. Both β-GaSe and ε-GaSe exhibit direct band gaps at the Γ point. For β-GaSe, the effective masses of electron ($\:{m}_{e}^{*}$) and light and heavy holes ($\:{m}_{lh}^{*}$ and $\:{m}_{hh}^{*}$) are $\:{m}_{e}^{*}$ = 0.2×m_0_, $\:{m}_{lh}^{*}$ = 0.13×m_0_ and $\:{m}_{hh}^{*}$ = 1.8×m_0_, respectively, where m_0_ is the free electron mass. In comparison, ε-GaSe has the effective masses of $\:{m}_{e}^{*}$ = 0.17×m_0_, $\:{m}_{lh}^{*}$ = 0.17×m_0_, and $\:{m}_{hh}^{*}$ = 1.7×m_0_, respectively. 

**Fig. 2:**
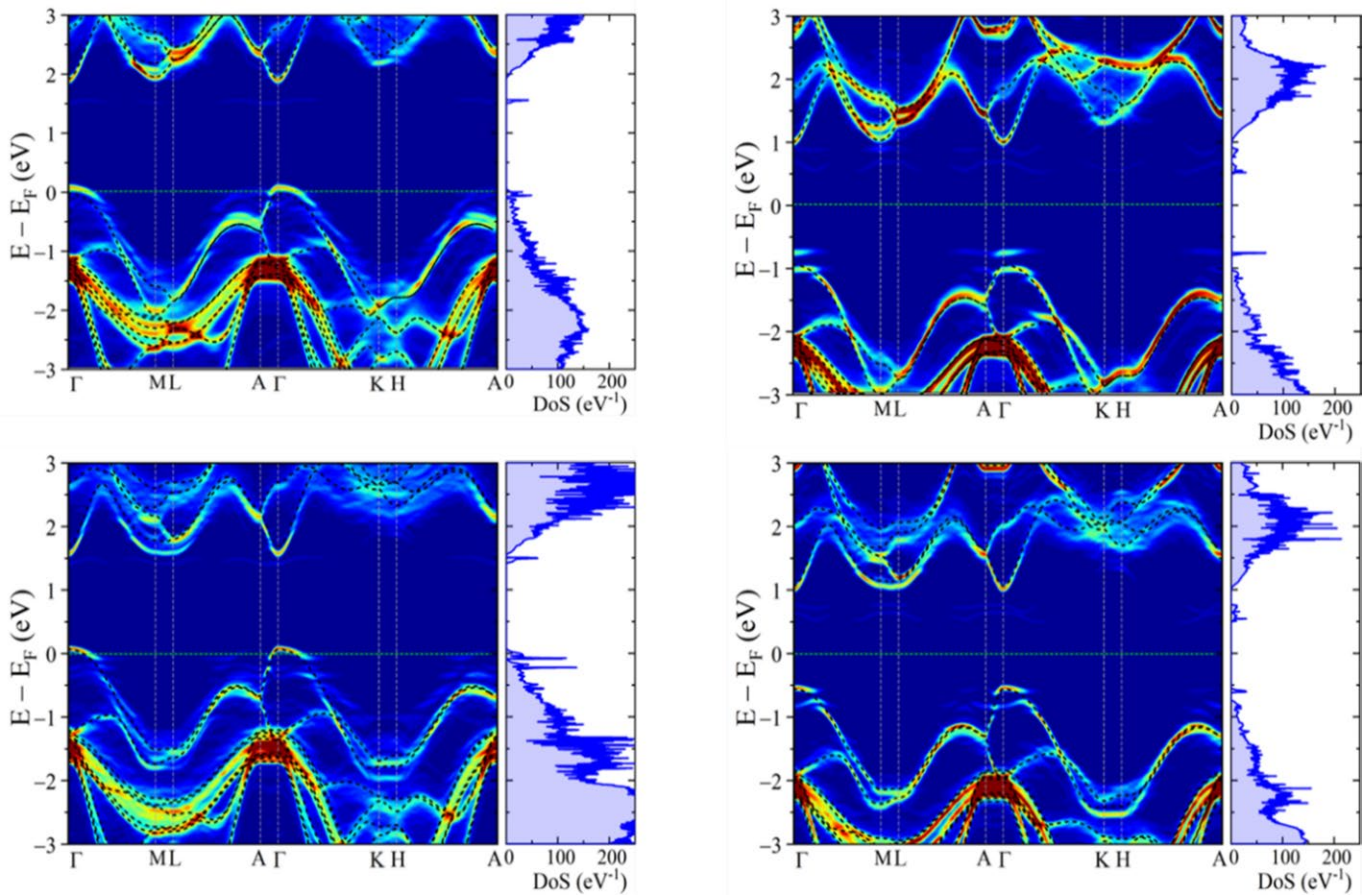
Unfolded band structures and density of states (DoS) for GaSe containing point defects. The first row corresponds to β-GaSe, and the second row to ε-GaSe. In each case, the left panel shows the electronic structure for a gallium vacancy (V_Ga_), while the right panel shows results for a selenium vacancy (V_Se_). The band structure plots display the unfolded spectral weight, where the colour intensity reflects the contribution of the defect supercell states to the primitive cell Brillouin zone. Red and blue indicate the two ends of the spectral weight spectrum, respectively. Darker shades signify minimal changes in the bands relative to the intrinsic structure without defects, indicating a significant overlap. Dashed black lines indicate the band structure of the corresponding pristine system for reference. The vertical axis shows energy relative to the Fermi level (E_F_ = 0 eV). Adjacent DoS plots illustrate the total density of states, presented in arbitrary units, revealing the presence and energy position of defect-induced states within the bandgap and near band edges

It is known that point defects – as inevitable structural features of grown 2D materials – create energy states in the material bandgap; hence, here we report the impact of Ga- and Se-vacancy on the electronic structure of GaSe (Fig. 2). Point defect calculations were carried out using a 4 × 4 × 2 supercell containing a single defect, corresponding to a defect density of approximately 1.6 × 10 cm^−3^. To model the defect, a lattice atom was substituted with an “empty” atom, a basis set of orbitals without any electron population effectively simulating a vacancy. As the supercell size increases, the corresponding first Brillouin zone proportionally contracts, resulting in significant band folding into the reduced Brillouin zone. This folding mask the original dispersion relations of the primitive cell, complicating the interpretation of defect-induced features in the band structure. To overcome this, we applied the band unfolding technique [53–55], which projects the supercell eigenstates onto the Brillouin zone of the primitive cell, thereby recovering the Bloch character of the electronic states. This approach enables a direct and meaningful comparison between the defected and pristine systems. When employing a linear combination of atomic orbitals (LCAO) basis set, unfolding proves especially effective in systems with structural perturbations, such as vacancies or impurities. It also reveals symmetry-breaking effects and provides valuable insight into how local disruptions influence the global electronic structure.

The unfolded band structure of systems containing gallium vacancies (V_Ga_) reveals their acceptor-like behaviour, as evidenced by the emergence of deep states within the valence band and a downward shift of the Fermi level. In contrast, selenium vacancies (V_Se_) act as donors, shifting the Fermi level upward and introducing shallow defect states near the conduction band edge. Both the β- and ε-GaSe polytypes exhibit similar qualitative responses to intrinsic defects; however, subtle differences are observed in the position of defect levels, the curvature of the bands, and the degree of hybridization between defect states and the valence or conduction band edges.

### Elemental analysis of freshly exfoliated GaSe

**Fig. 3:**
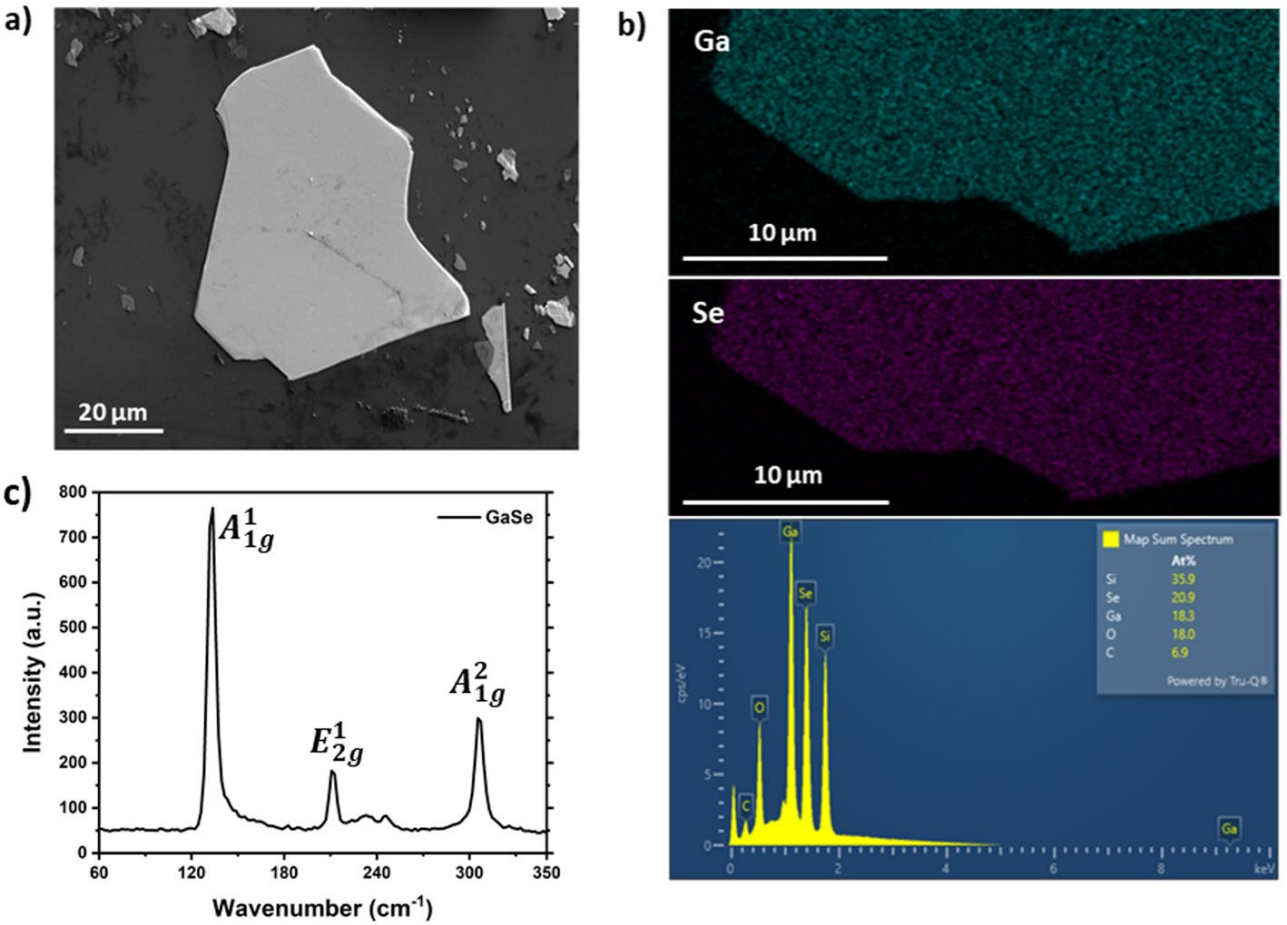
**a** SEM image depicting a GaSe flake that has been exfoliated onto an 85 nm SiO_2_/Si substrate. **b** EDX data of the GaSe flake showing Ga and Se maps, followed by map spectrum. **c** Raman spectrum of a freshly exfoliated GaSe flake recorded using a 532 nm laser

Figure 3a presents typical scanning electron microscopy (SEM) images of the 2D GaSe flake. The SEM images display well-defined GaSe flakes characterized by sharp edges, which indicate a clean exfoliation process. The surface is relatively smooth, exhibiting minimal contamination. Step edges and terraces can be observed in certain flakes, implying variations in thickness across the flake. Although SEM does not directly quantify thickness, the differences in contrast and the presence of terrace steps offer qualitative insights. Thinner areas are depicted with lower contrast, whereas thicker regions exhibit greater brightness due to an increased secondary electron yield. EDX (Energy-Dispersive X-ray Spectroscopy) results for a 2D GaSe flake, as illustrated in Fig. 3b, reveal the presence of gallium (Ga) and selenium (Se) as the primary elements. The overview of the GaSe flake (see Fig. 3b) indicates the following elements in atomic percentage: Si (35.9%), Se (20.9%), Ga (18.3%), O (18.0%), and C (6.9%).

The Raman spectrum of GaSe was measured using 532 nm excitation, as shown in Fig. 3c. The GaSe Raman peaks are identified at 133.6, 212.5, and 307.2 cm^−1^ (Fig. 3c), corresponding to the A^1^_1g_, E^1^_2g_, and A^1^_2g_ vibrational modes of GaSe, respectively [56]. The most prominent A^1^_1g_ peak arises from out-of-plane vibrations, while the other peaks pertain to in-plane modes. The measured distance between the out-of-plane modes at 173.6 cm^–1^ is characteristic of that found in bulk and few-layer GaSe, which is approximately 173 cm^–1^. Under ambient conditions, GaSe flakes undergo photo-oxidation and this occurrence can be observed by tracking the decrease in intensity of the GaSe Raman peaks and the emergence of additional peaks linked to oxidation products like Ga_2_Se_3_, Ga_2_O_3_, and amorphous selenium, which we discuss in the next section.

### Analysis of the properties of ‘aged’ GaSe

GaSe in both its film and exfoliated form is known to degrade in ambient air over time, similar to other selenide compounds [57, 58]. This degradation can have irreversible effects on the materials structure, which in turn affect its optical and electrical properties [59]. Understanding how the degradation occurs and how to measure it are key to developing methods to protect the material to ensure that any devices made from GaSe can be optimised while maintaining its unique properties over a longer period of time.

**Fig. 4:**
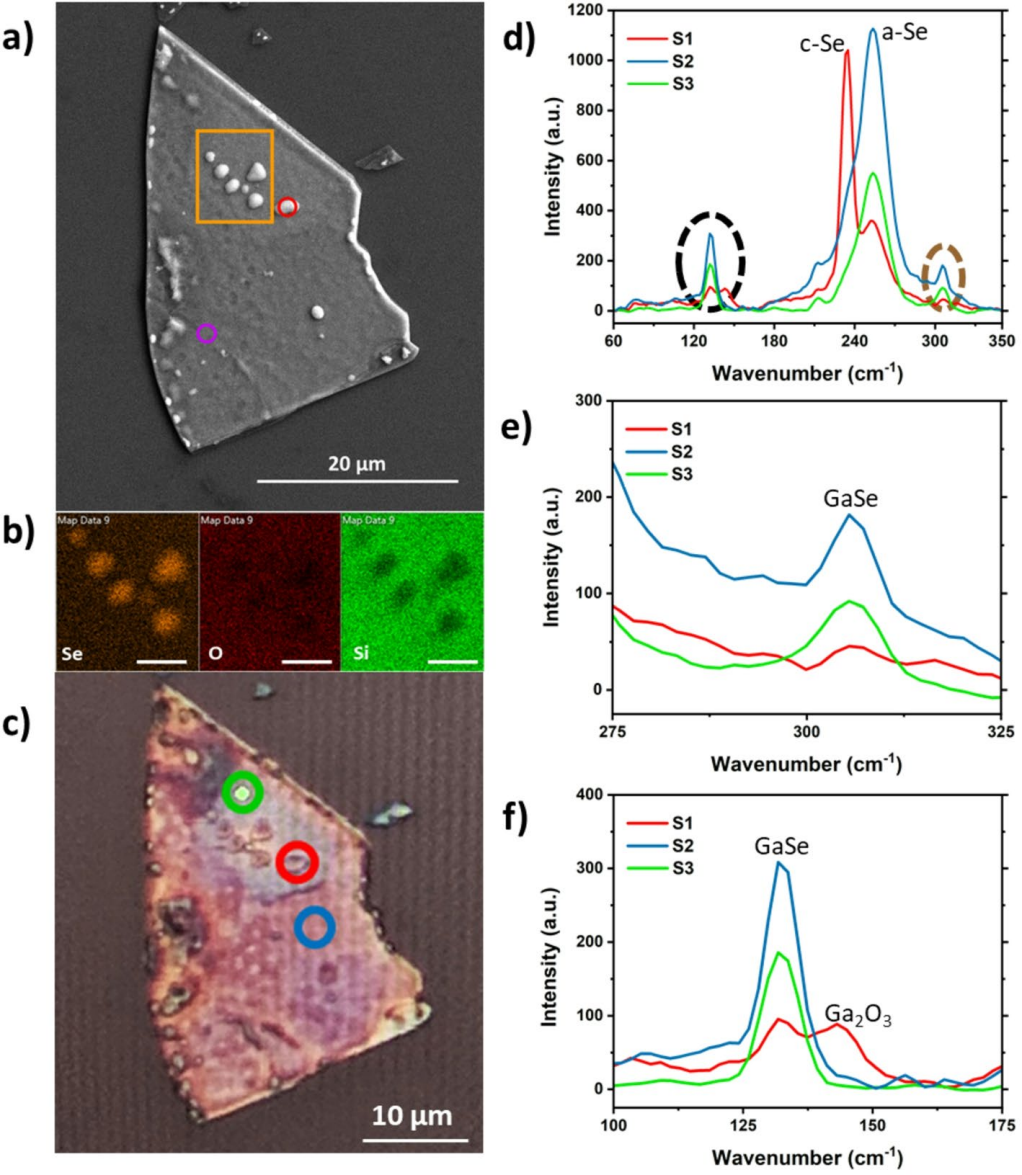
**a** SEM image of ~ 6-month-old exfoliated GaSe flake with the EDX mapping area shown by the orange box. The purple and red circle indicate where a point scan was taken during the EDX. **b** EDX elemental maps from left to right of Se, O and Si highlighting the Se-rich blisters on the material surface. The scale bar is 2.5 μm. **c** Optical microscope image at x50 of the flake as seen in (**a**), showing the three areas scanned with Raman spectroscopy. The red circle represents (S1), blue circle (S2) and green circle (S3) in spectra (**d**–**f**). **d** Raman spectra of the three scan areas. **e **and **f** Zoomed in Raman spectra of the brown and black dashed regions respectively as shown in (**d**)

The degradation of GaSe occurs mainly in its topmost layers through the oxidation of Ga to Ga_2_O_3_ and the formation of Se-rich particles and Ga_2_Se_3_ [46]. The Se-rich particles, which present as hemispherical protrusions (blisters) on the materials surface can be seen in Fig. 4a and in the EDX mapping of Fig. 4b. The EDX analysis indicates that the blisters are indeed Se-rich as both the oxygen and silicon signals are deficient in the scan area in contrast to that of the selenium signal. The EDX point scans of the ~ 6-month-old flake shown in Fig. 4a, represent a Ga/Se ratio of 0.38 and 1.58, as highlighted by the red and blue circles, respectively. The ideal ratio for Ga/Se in this material is 1, strongly suggesting that the ratio of 0.38 at the blister is Se-rich, while the ratio of 1.58 taken on a blister-free region, appears to be Se-deficient.

To further understand the reaction occurring on the flake, Raman spectroscopy was also performed. As highlighted in the optical microscope image in Fig. 4c, three regions were scanned, represented by the green, red and blue circles. This analysis includes that of the blister that was analysed with EDX again represented by the red circle. The results seen in Fig. 4d demonstrate the dominant GaSe Raman vibrational modes A_1g_^1^ 132.59 cm^− 1^, E_2g_^1^ 211.53 cm^− 1^ and A_1g_^2^ 305.48 cm^− 1^. However, there are additional peaks present, including 143.21 cm^− 1^ which is indicative of Ga_2_O_3_ provided in more detail in Fig. 4e as well as two intense peaks located at 234.04 cm^− 1^ and 253.92 cm^− 1^ signifying crystalline (c-Se) and amorphous (a-Se) selenium, respectively. The c-Se vibrational mode can only be seen on the blister region, while the a-Se appears both in the blister and the surrounding region.

**Fig. 5:**
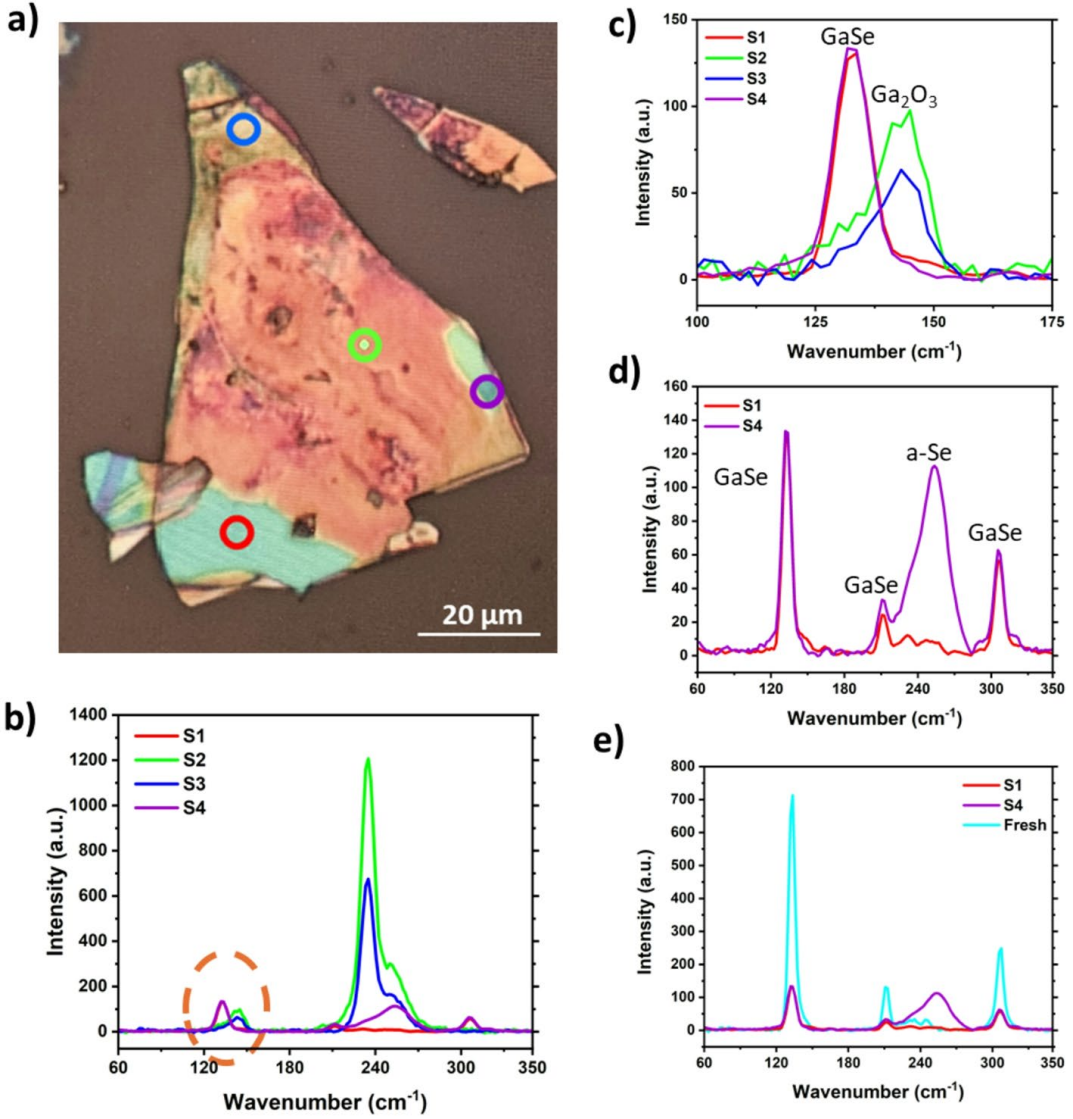
**a** Optical microscope image at x50 of an exfoliated GaSe flake aged for ~ 6 months with indicated Raman scan areas. The red circle represents (S1), green circle (S2), blue circle (S3) and purple circle (S4) for the plots (**b**)–(**d**).** b** Raman spectra of all four scan areas. **c** Zoomed in Raman spectra of the orange dashed region seen in (**b**).** d** Raman spectra of the areas marked by the red and purple circles. **e** Raman spectra of the areas marked by the red and purple circles with the additional spectrum taken from a freshly exfoliated flake (light blue)

A second exfoliated flake, aged for ~ 6 months and prepared and exposed under the same conditions as the previous sample, was analysed by Raman spectroscopy, as shown in Fig. 5a. This flake had no signs of blisters present but appeared to have a number of distinct regions of various colours, each of which were measured and marked with a coloured circle. Figure 5b is a combination of all four scan areas. The scan areas highlighted by the green and blue circles have two noticeable peaks at 234.92 cm^− 1^ and 250.8 cm^− 1^ which similar to the flake in Fig. 4a depicts c-Se and a-Se, respectively. Interestingly, there are no GaSe peaks observed. Figure 5b shows the GaSe A_1g_ 132.6 cm^− 1^ peak as well as the Ga_2_O_3_ peak at 143.5 cm^− 1^, highlighting how the different regions of the flake are at different stages of degradation. Figure 5d shows the scan areas marked by the red and purple circles, which show comparable vibrational modes of lower intensity. However, Fig. 5e makes it apparent that these scan areas are much closer to the pristine exfoliated crystal than the other regions on the flake. There is one peak at 252.74 cm^− 1^ representing a-Se, while all other peaks observed align with the dominant Raman vibrational modes for GaSe albeit reduced intensity.

The results strongly indicate the degradation of GaSe over time forming Ga_2_O_3_ as well as c-Se and a-Se. It is evident from the EDX data that the blisters forming at the surface over time are Se-rich and this is further supported by the Raman spectra. These results demonstrate that the degradation is not inherently uniform across the material. This is especially evident when the same procedure and air-exposure produce different results, as not all the material will form visible blisters at the surface and other flakes appear to degrade at different rates which is made clear through the Raman data obtained. This is in contrast to the uniform degradation found on other flakes of the same material as seen in Fig. S2, which further demonstrates how this material has variable degradation when subjected to the same conditions and time period.

## Conclusion

The 2D material GaSe has multiple polytypes with β-GaSe being the most common as it is thermodynamically stable at room temperature. This polytype has a highly symmetrical, hexagonal layered structure the effects of which give this polytype a 2 eV bandgap as demonstrated through DFT analysis and supported with absorption spectrum measurements. SEM, EDX, and Raman spectroscopy analyses of fresh and approximately 6-month-aged flakes allowed the determination of the effects of air exposure on exfoliated GaSe. This was especially evident from the hemispherical, Se-rich blisters produced as a result of Se-Se bonds caused by the oxidation of Ga forming Ga_2_O_3_. This oxidation is most likely a result of point-defects on the materials surface as well as dangling bonds caused from exfoliation leading to oxygen absorption sites. The Se-rich blisters were identified with EDX analysis but confirmed with Raman showing that they contained both amorphous and crystalline (trigonal) phases of Se. Although the blisters were not present on every sample, highlighting the varied nature of GaSe degradation, the Raman analysis suggests that the oxidation alters the materials structure leading to a Se-rich characteristic of the material. This was observed from the clear evidence of both Se phases in the regions of the flakes where no blisters were present. It is also important to note that these phases were observed irrespective of whether the flake degraded uniformly or not over the ~ 6-month aging period. The outcomes learned from this study are applicable to various types of 2D materials as they highlight the importance of oxidation and its effects on the surface chemistry and stability within these materials. The oxidation and Se-rich characteristic in the aged material could produce additional applications for these materials in the future.

## Methods

All the GaSe flakes were mechanically exfoliated from their bulk crystal using the Scotch tape technique. The flakes on the scotch tape were then gently pressed Si/SiO_2_ (85 nm) substrates. Once exfoliated, all samples were stored in a cleanroom with controlled relative humidity of 45.3% and temperature of 21.5 °C for ~ 6 months. A Tescan Amber X FIB-SEM with Oxford Ultim Max Energy Dispersive X-ray Spectroscopy (EDX) was used for surface and elemental characterization. The energy of the beam was set to 5 keV and the BC was 1 nA to properly detect all the features of the surface. Raman spectra were acquired with a Horiba XploRA™ Plus confocal Raman microscope equipped with a 70 mW, 532 nm laser. All the Raman spectra, if not otherwise specified, were acquired with a power of 0.5%, with an acquisition time of 10 s and an accumulation time of 10 s, using a 50x objective. Raman data handling and processing were carried out in Origin. Raw data were baseline-corrected using a spline interpolation method in OriginPro2022b. Anchor points were manually placed at baseline regions between peaks to model and subtract the background signal. To avoid overfitting of the data, deconvolution of the spectra was only performed on the most prominent bands A_1g_^1^ (132 cm^−1^), E_2g_^1^ (211 cm^−1^), and A_1g_^2^ (305 cm^−1^) for GaSe, B_2g_ (143 cm^−1^) for Ga_2_O_3_, and c-Se (235 cm^−1^) and a-Se (250 cm^−1^) for Se.

Spectral response measurements were made using a Bentham PVE300 spectral response kit. This includes lamps, diffraction grating, bandpass filter and exit slits to illuminate the sample with about 1 nm spectral resolution, with an optical power on the order of 20 uW. The Bentham chopper was disconnected, and illumination was continuous wave. Optical spot size was approximately 3 mm square, much larger than the flake under test, and makes the estimate of power intercepted by the flake difficult to determine. Gold contact pads to the flake were probed, and DC photocurrent characteristics recorded using a Keithley 2400 source meter in high resolution mode for different applied voltages. For a fixed voltage, the illumination wavelength was stepped in 2 nm increments from 350 to 800 nm.

First-principles electronic structure calculations were conducted using density functional theory (DFT) within the generalized gradient approximation (GGA), as implemented in QuantumATK [60]. Norm-conserving pseudopotentials were employed to describe the exchange–correlation potential. OpenMX numerical atomic orbital basis sets were used, with configurations of *s*2*p*2*d*2*f*1 for Ga atoms and *s*2*p*2*d*1 for Se atoms [61]. Brillouin-zone sampling was performed using the Monkhorst–Pack scheme, with a k-point grid density of approximately 10 k-points/Å [62]. Real-space integrals were computed using an energy cutoff of 210 Rydberg. It is well established that standard DFT calculations tend to underestimate the quasiparticle bandgap. To address this limitation, approximate quasiparticle corrections such as those used in the GGA-1/2 method have been shown to significantly improve bandgap estimations [63, 64]. In this approach, a self-energy correction potential is introduced at the atomic level to compensate for the electron–hole self-interaction. This correction is derived from the difference between the potential of a neutral atom and that of a partially ionized atom, created by removing a fraction of its electronic charge. Notably, the GGA-1/2 method offers a considerable computational advantage over hybrid functional approaches, making it an efficient alternative for bandgap correction. The atomic geometries were fully relaxed until the residual forces on each atom were less than 0.01 eV/Å.

## Supplementary Information

Below is the link to the electronic supplementary material.

Supplementary Material 1

## Data Availability

Data sets generated during the current study are available from the corresponding author on reasonable request.
