# Supplementary material for: Air sensitivity of GaSe 2D material and its potential implications on device reliability
Source: Discov Nano. 2026 Apr 17;21(1):126. doi: 10.1186/s11671-026-04568-9 (PMC13090464; doi:10.1186/s11671-026-04568-9)
Supplement: Supplementary file 1 — Supplementary Material 1 [file 11671_2026_4568_MOESM1_ESM.docx]

**Supplementary Information**

**Air sensitivity of GaSe 2D material and its potential implications on device reliability**

**Hazel Neill^1,*^, Lida Ansari^1^, Vilas Patil^1^, Stephen O’Sullivan^1^, Brendan Roycroft^1^, Martina Piletti^1^, Daniela Iacopino^1^, Paul K. Hurley^1,2^, Farzan Gity^1,*^**

^1^ Tyndall National Institute, University College Cork, Lee Maltings, Dyke Parade, Cork, T12 R5CP, Ireland

^2^ School of Chemistry, University College Cork, Cork, Ireland

^*^Corresponding authors: hazel.neill@tyndall.ie & farzan.gity@tyndall.ie


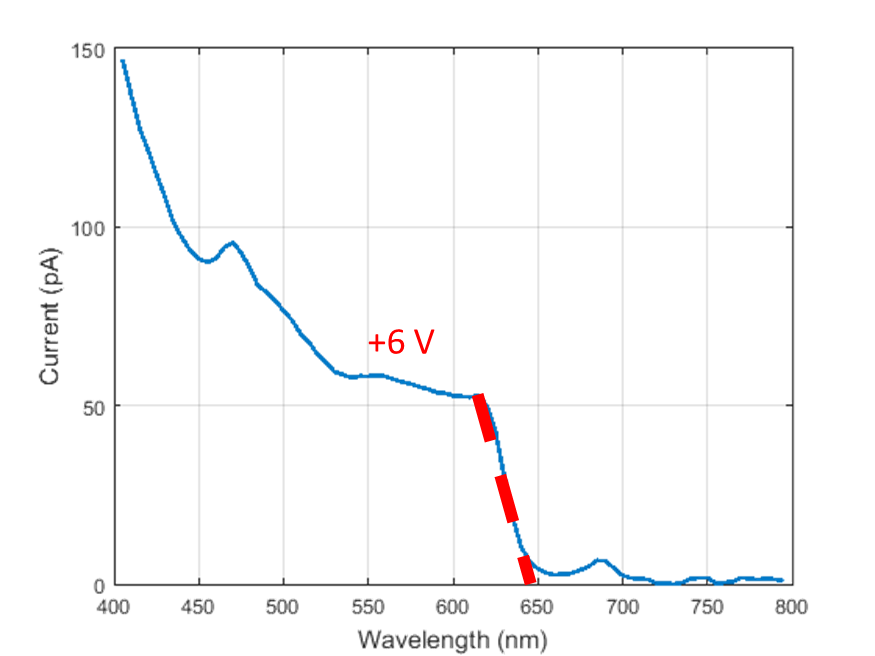
 The absorption spectrum measurements were taken by probing the source and drain contacts with a Keithley 2400 source meter and applying +6 V bias while sweeping the wavelength from 400-800 nm in 10 nm increments.

**Figure S1**: Absorption spectrum of GaSe bulk crystal used, showing the edge of the bandgap to be 645 nm which corresponds to an approximately 1.92 eV bandgap, indicating that the crystal is the β-polytype.


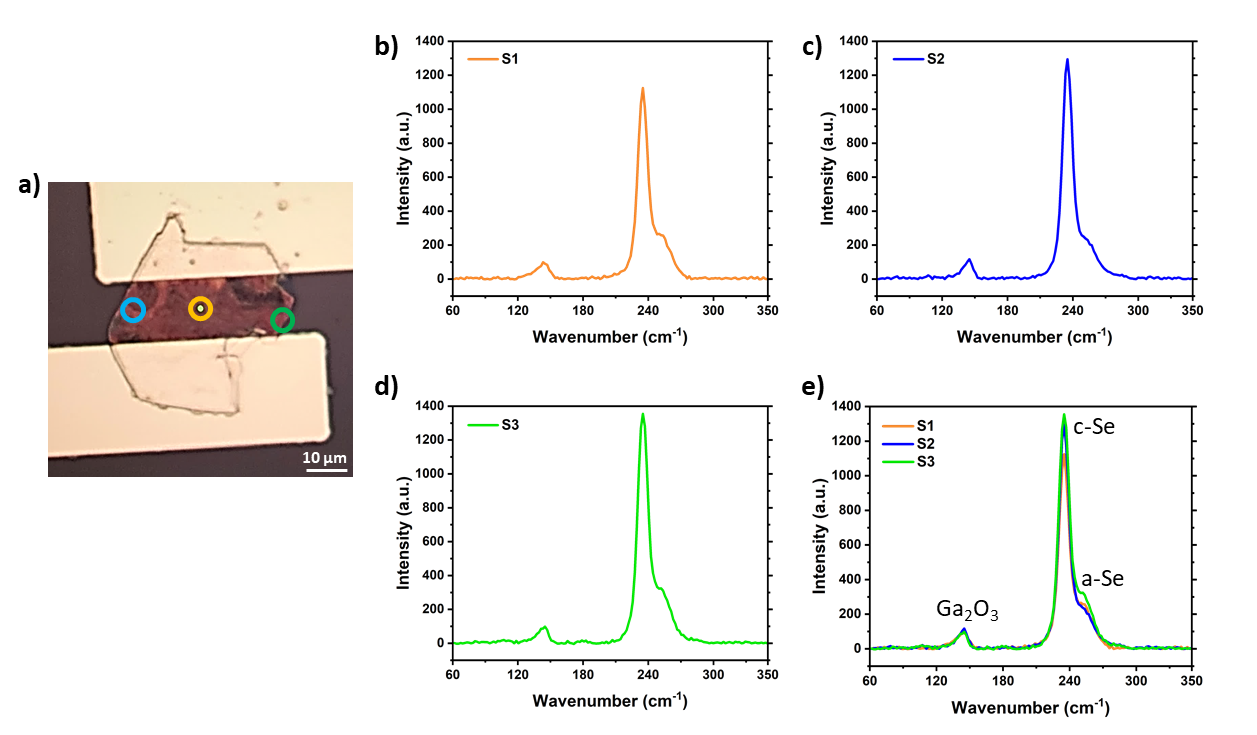


**Figure S2:** **a)** Optical microscope image of an aged flake showing three Raman scan areas with the orange circle representing S1, blue circle S2 and green circle S3. **b)-d)** Individual Raman spectra and **e)** combined Raman spectra of the scanned areas.

It is evident from these results, that the degradation occurring across the exposed area of the flake is uniform. This can be seen from the three vibrational modes present for each point at 143.9 cm^-1^, (Ga_2_O_3_ peak) 235.19 cm^-1^ (c-Se peak) and 252.91 cm^-1^ (a-Se peak) with similar intensities for each scan point. The dominant vibrational modes for GaSe at approx. 132, 211 and 305 cm^-1^ are absent across the material.
